# Obesity is associated with changes in oxysterol metabolism and levels in mice liver, hypothalamus, adipose tissue and plasma

**DOI:** 10.1038/srep19694

**Published:** 2016-01-22

**Authors:** Owein Guillemot-Legris, Valentin Mutemberezi, Patrice D. Cani, Giulio G. Muccioli

**Affiliations:** 1 Bioanalysis and Pharmacology of Bioactive Lipids Research Group, Louvain Drug Research Institute, Université catholique de Louvain, Belgium; 2 Metabolism and Nutrition Research Group, WELBIO- Walloon Excellence in Life Sciences and BIOtechnology, Louvain Drug Research Institute, Université catholique de Louvain, Brussels, Belgium

## Abstract

Oxysterols are bioactive lipids derived from cholesterol that are linked to inflammatory processes. Because obesity and metabolic syndrome are characterized by inflammation and altered cholesterol metabolism, we sought to investigate the variations of oxysterol levels and their metabolic pathways induced by obesity in the liver, hypothalamus, adipose tissue and plasma. To this end, we used diet-induced and genetic (*ob/ob* and *db/db*) models of obesity. Among the oxysterols measured, we found that 4β-oxysterol levels were consistently decreased in the high-fat diet study, at different time-points, and in the *ob/ob* model. Overall, we did not find any correlation between cytochromes mRNA expression and variations of oxysterol levels. We also measured the levels of hepatic primary bile acids, in these three models and found similar profiles between HFD and *ob*/*ob* mice. However, although they are downstream metabolites of oxysterols, the variations in bile acid levels did not reflect the variations of their precursors. Our data show that, when considering oxysterol metabolism, the high-fat diet and *ob/ob* models are more closely related when compared to the *db/db* model. However, we were able to discriminate between lean and obese phenotypes based on liver oxysterol (4β-hydroxycholesterol, 27- hydroxycholesterol, 7-hydroxycholestenone) levels and enzyme (CYP3A11, CYP27A1, CYP7A1) expression.

Obesity is associated with comorbidities collectively known as the metabolic syndrome and represents one of the most alarming health issues of modern times^1^,^2^,^3^. Amongst other etiologies, a high-fat diet^4^,^5^,^6^ and cholesterol metabolism are key players in the genesis of these disorders^7^,^8^. Accordingly, a low HDL cholesterol level is one of the five criteria clinically used to diagnose the presence of metabolic syndrome^9^,^10^. In this context, changes in cholesterol synthesis during obesity have been well studied, including as a means of treatment^11^. In contrast, the pathways leading to cholesterol catabolism are less documented. This is especially the case for the formation of oxidized derivatives of cholesterol, better known as oxysterols.

Oxysterols can be formed through enzymatic reactions, mainly involving the CYP 450 enzymes super-family, or they can be formed through non-enzymatic pathways^12^,^13^,^14^. Their metabolism has been mainly documented in humans and only to a lesser extent in mice where the metabolic pathways of oxysterols are only partially characterized. These bioactive lipids were once considered as mere by-products of cholesterol catabolism, but nowadays their involvement in apoptosis or inflammatory processes^15^,^16^,^17^,^18^, as well as in conditions such as atherosclerosis or Alzheimer’s disease has been demonstrated^13^,^19^. Furthermore, the central role played by several oxysterols as regulators of cholesterol metabolism, upon liver X receptors (LXR) binding, is now well characterized^20^,^21^,^22^.

In light of the emerging role of these bioactive lipids in inflammation and metabolic syndrome^23^,^24^, we decided to study oxysterol levels and the expression of the main enzymes responsible for their metabolism in several mice models of obesity and type 2 diabetes. Here, we first focused our attention on the liver and the hypothalamus as they play key roles during obesity. Indeed, on the one hand, the liver is the main organ responsible for the organism’s cholesterol metabolism and liver homeostasis is deeply affected by obesity^25^. On the other hand, the hypothalamus is the integration center of food intake and is also affected by obesity. Indeed, obesity is associated with a disruption of the homeostasis in this part of the central nervous system, generating changes in the inflammatory tone and modifying the expression of several neuro-peptides involved in food intake^26^,^27^. To try to explain the changes we observed in the liver and hypothalamus, we also quantified the oxysterol levels in the plasma and in the adipose tissue as the latter may serve as storage compartment. Finally, we also quantified the primary bile acids as they represent the final step in cholesterol and oxysterol metabolism.

## Results

### Obese phenotype induced by the high-fat diet

In order to study the effect of obesity development on the oxysterol levels, we randomly assigned 96 mice to either a control group (8 mice/group) or a diet-induced obesity group (8 mice/group) and fed them with normal diet (control) or a high-fat diet (diet-induced obesity) for 1 week, 2 weeks, 4 weeks, 8 weeks or 16 weeks. At each time-point, a control group and a high-fat diet group were sacrificed and studied. To characterize the obese phenotype induced by high-fat feeding, we performed time-domain NMR before sacrifice to quantify fat and lean masses^28^. After 1 week of high-fat feeding mice already show a significant increase in weight as well as in their fat mass (Fig. SI 1a,b). As expected, the groups sacrificed after longer periods of high-fat feeding display an increased weight and fat mass (Fig. SI 1a,b). To clearly illustrate the presence of a hepatic steatosis after 16 weeks of high-fat diet, we measured the lipid content in the liver and found it to be 3-fold higher than in control animals (Fig. SI 1d), this result was confirmed by histological analysis (Fig. SI 1c). We also found increased plasmatic cholesterol, triglyceride and glucose contents in mice fed a high-fat diet compared to their respective controls at week 16 (Fig. SI 1g). Invasion of the tissues by macrophages is a characteristic of prolonged high-fat diet feeding. Here we found that the mRNA expression of F4/80, a myeloid lineage marker expressed by macrophages or microglial cells, was significantly increased after 16 weeks of high-fat diet in the liver (Fig. SI 1e) and in the hypothalamus (Fig. SI 1f). To further characterize the inflammatory tone induced by the high-fat diet, we measured the expression of interleukin-6 (IL-6) and toll-like receptor 4 (TLR4) in the liver and found both markers to be increased in the high-fat diet group at week 16 (Fig. SI 1e). As for the hypothalamus, the inflammatory tone at week 16 is further demonstrated by the increased expression of IL-6 and two markers of astrocytes activation i.e. glial fibrillary acidic protein (GFAP) and Serpina3n (Fig. SI 1f). These last two markers indicate both an activation of astrocytes as well as astrogliosis which are associated with the inflammation induced by a high-fat diet^29^,^30^.

### Hypothalamic oxysterol metabolism during a high-fat diet

**Figure 1:**
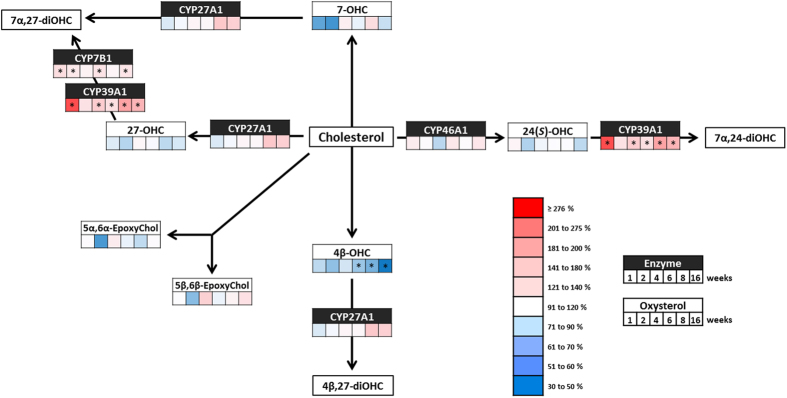
Hypothalamic oxysterol levels and expression of their metabolic enzymes during the development of diet-induced obesity. Schematic representation of the main metabolic steps involved in the synthesis and degradation of the oxysterols measured throughout the HFD study in the hypothalamus. The figure shows the variations in oxysterol levels and mRNA expression of their metabolic enzymes, compared to the respective control mice, at the different time-points throughout the study. At each time-point (i.e. 1; 2; 4; 6; 8 and 16 weeks) a normal diet (control) and a high-fat group were sacrificed. Oxysterol levels were quantified by HPLC-MS and mRNA enzyme expression was measured by qRT-PCR. Red color indicates an increase and blue color indicates a decrease of the HFD group compared to control group. Data (mean ± s.e.m) are reported in Table S1. Student’s t-test between HFD group and the respective CTL group. *P < 0,01.

Next, we quantified oxysterols in the hypothalamus using a validated HPLC-MS method^31^. We first looked at 24(*S*)-hydroxycholesterol (24(*S*)-OHC) as it is the most abundant oxysterol in the central nervous system^32^ (Fig. SI 3b). We found no significant modification of its levels throughout our study (Fig. 1 and Table S1). 24(*S*)-OHC is synthesized by CYP46A1 and is then either exported to the circulation or transformed into 7α,24-dihydroxycholesterol (7α,24-diOHC) by CYP39A1^33^,^34^. In our time-course study, the high-fat diet did not affect CYP46A1 mRNA expression, but significantly increased the expression of CYP39A1 already after 1 week (Fig. 1 and Fig. SI 2a). The levels of 27-hydroxycholesterol (27-OHC), an oxysterol oxidized on the side-chain similarly to 24(*S*)-OHC, were not affected by the diet at any of the selected time-points. Concerning the oxysterols oxidized on the sterol ring, we noted a significant decrease of 7-hydroxycholesterol (7-OHC) levels for mice fed a high-fat diet at week 1 and week 2 followed by a normalization at the following time-points (Fig. 1 and Table S1). 7-OHC can be formed through ROS dependent oxidation or through an enzymatic pathway involving CYP7A1^35^,^36^. However, we could not detect any expression for CYP7A1 in the hypothalamus, consistent with its reported exclusive hepatic expression^36^. 7-OHC is thought to be further metabolized by CYP27A1, the expression of which is not affected by the high-fat diet until later time-points (weeks 8 and 16) (Fig. 1 and Fig. SI 2a). The evolution of 5α,6α-epoxycholesterol and 5β,6β-epoxycholesterol levels in the high-fat fed mice follow similar patterns although the variations do not reach statistical significance (Fig. 1 and Table S1). Thus, the most significant variations observed in the hypothalamus were found for 4β-hydroxycholesterol (4β-OHC). Indeed, the levels do not differ from control’s until week 4 and then significantly decrease from week 6 to reach 40% of the control levels at week 16 (Fig. 1 and Table S1). The enzyme responsible for 4β-OHC synthesis in mouse central nervous system is yet to be identified. Indeed, in humans CYP3A4 or CYP3A5 are responsible for 4β-OHC production, but the respective murine ortholog CYP3A11 and CYP3A13^37^,^38^ are not expressed in the hypothalamus. Similarly to 7-OHC, 4β-OHC is enzymatically catabolized by CYP27A1, the expression of which is increased in the later time-points of our study (Fig. 1 and Fig. SI 2a). This could partly explain the decrease of 4β-OHC levels at the same time-points. Of note, besides hydroxylation, the elimination of oxysterols from the central nervous system is favored by the introduction of a sulfate group on the 3-hydroxyl position by the cholesterol sulfotransferase (SULT2B1b)^39^,^40^. In our study, this enzyme does not show any modification of its expression in the hypothalamus (Fig. SI 2a).

### Hepatic oxysterol metabolism during a high-fat diet

**Figure 2:**
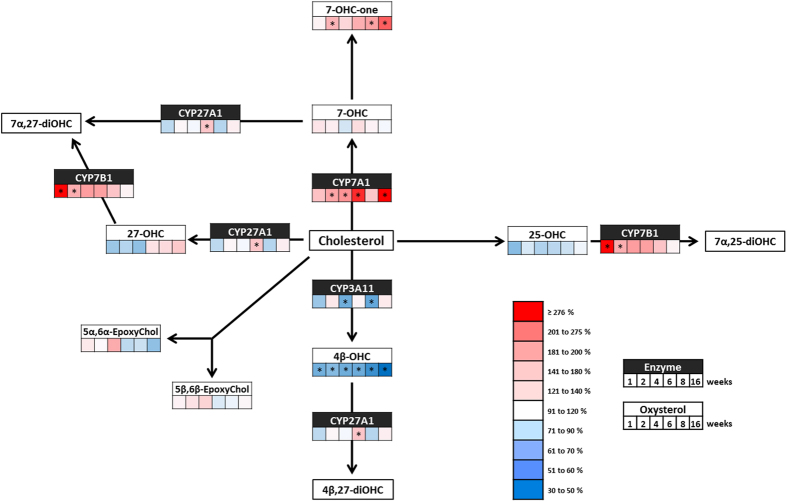
Hepatic oxysterol levels and expression of their metabolic enzymes during the development of diet-induced obesity. Schematic representation of the main metabolic steps involved in the synthesis and degradation of the oxysterols measured throughout the HFD study in the liver. The figure shows the variations in oxysterol levels and mRNA expression of their metabolic enzymes, compared to the respective control mice, at the different time-points throughout the study. At each time-point (i.e. 1; 2; 4; 6; 8 and 16 weeks) a control and a high-fat group were sacrificed. Oxysterol levels were quantified by HPLC-MS and mRNA enzyme expression was measured by qRT-PCR. Red color indicates an increase and blue color indicates a decrease of the HFD group compared to control group. Data (mean ± s.e.m) are reported in Table S2. Student’s t-test between HFD group and the respective CTL group. *P < 0,01.

4β-OHC is the most abundant oxysterol in the liver (Fig. SI 3a). Here, we found that the high-fat diet causes a rapid decrease in the levels of this oxysterol, decrease that was maximal after 16 weeks of high-fat feeding (Fig. 2 and Table S2). However, mRNA expression of both CYP3A11 and CYP27A1, the enzymes responsible for its production and degradation, was not affected by the diet (Fig. 2 and Fig. SI 2b). The levels of the two epoxycholesterols, 5α,6α-epoxycholesterol and 5β,6β-epoxycholesterol, as well as those of 25-hydroxycholesterol (25-OHC) were not affected by the high-fat diet. Conversely, the levels of 7α-hydroxy-4-cholesten-3-one (7α-OHCnone), a key intermediate in the synthesis of bile acids, are increased by the high-fat diet feeding (Fig. 2 and Table SI 2). In human, plasma levels of 7α-OHCnone are reported to correlate with hepatic CYP7A1 mRNA expression^41^,^42^ and interestingly we found an increased expression of CYP7A1 mRNA (Fig. 2 and Fig. SI 2b) in the liver during our time-course study allowing us to establish a correlation between hepatic 7α-OHCnone levels and the expression of CYP7A1 (Pearsons’ r = 0,66; p-value < 0,0001).

Because oxysterols are cholesterol derivatives, we wondered whether there is a correlation between the hepatic cholesterol and oxysterol levels. The analysis of the data did not reveal any such correlation (data not shown). Of note, when the oxysterol levels are normalized to the cholesterol content, rather than to the tissue weight, similar variations induced by the high-fat diet were found (Table S3).

### Obesity alters oxysterol levels in the plasma and the adipose tissue

**Figure 3:**
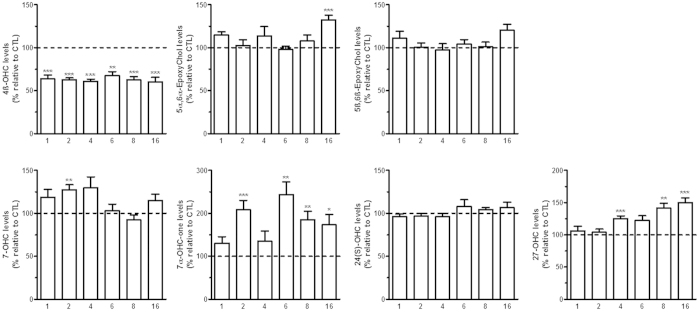
Plasmatic oxysterol levels during the development of diet-induced obesity. Oxysterol levels were quantified by HPLC-MS in mouse plasma of the diet-induced obesity model. At each time-point (i.e. 1; 2; 4; 6; 8 and 16 weeks) a control and a high-fat group were sacrificed. The data are reported as percentage of each respective control group (shown as a dotted line). Data are mean ± s.e.m. Student’s t-test between HFD group and the respective CTL group. *P < 0,05 and **P < 0,01 and ***P < 0,001.

We studied the oxysterol levels in the plasma at each selected time-point of the high-fat diet model. Interestingly, similarly to what we found in the liver and hypothalamus, we found a sustained decrease in the levels of 4β-OHC from week 1 to week 16 (Fig. 3). Furthermore, we measured an increase in the plasmatic levels of 7α-OHCnone and 27-OHC at the later time-points (Fig. 3). This is consistent with the increased levels of these two oxysterols found in the liver (Fig. 2).

**Figure 4:**
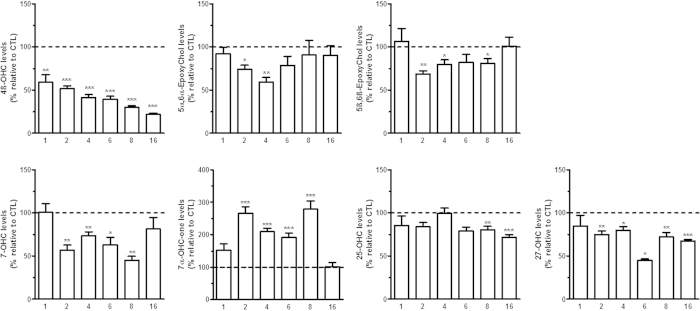
Adipose tissue oxysterol levels during the development of diet-induced obesity. Oxysterol levels were quantified by HPLC-MS in mouse subcutaneous adipose tissue of the diet-induced obesity model. At each time-point (i.e. 1; 2; 4; 6; 8 and 16 weeks) a control and a high-fat group were sacrificed. The data are reported as percentage of each respective control group (shown as a dotted line). Data are mean ± s.e.m. Student’s t-test between HFD group and the respective CTL group. *P < 0,05 and **P < 0,01 and ***P < 0,001.

Because, we observed a drastic decrease in the levels of 4β-OHC in the liver, hypothalamus and plasma, we wondered if this oxysterol could be stored in the adipose tissue as this tissue expands as soon as week one in the high-fat diet mice. Therefore, we set out to measure the oxysterol profile in this tissue. Surprisingly, we evidenced a marked and sustained decrease in 4β-OHC levels at each time-point (Fig. 4). Thus, the adipose tissue does not seem to store the 4β-OHC, in the settings of diet-induced obesity. Of note, we also measured opposite changes in the levels of 7-OHC and 7-OHCone. Indeed while we found decreased levels of 7-OHC at weeks 2, 4, 6 and 8 we found increased amounts of its metabolite 7-OHCone at the same time-points (Fig. 4). Finally, we measured a decrease at the two later time-points for adipose levels of 25-OHC and a decrease in adipose levels of 27-OHC at every time-points except at week 1 (Fig. 4). As for the liver, we found similar results when normalizing the data to the cholesterol levels (Table S3).

### Oxysterol metabolism in genetic models of obesity

Since oxysterol levels were altered during the course of the high-fat diet study, we wondered if similar variations could be observed in a genetic model of obesity, therefore we quantified oxysterols in hypothalami and livers of *db/db* and *ob/ob* mice. In the hypothalamus, similarly to the high-fat diet study, we found no variation in 24(*S*)-OHC levels between the *db/db* mice and their lean control (db/lean) littermates (Fig. SI 4a). However, we did not observe the decrease in 4β-OHC levels that was found in the high-fat diet mice. Another difference between the high-fat diet model and the *db/db* model is apparent when looking at the hypothalamic levels of 27-OHC which are decreased in the *db/db* mice compared to the db/lean (Fig. SI 4a). This decrease could be, at least in part, ascribed to an increased mRNA expression of CYP7B1 and CYP39A1 (Fig. SI 4a). Finally, we found a higher SULT2B1b mRNA expression in the hypothalamus of *db/db* mice compared to db/lean mice (Fig. SI 4a) suggesting an increased elimination of oxysterols through the sulfotransferase mechanism.

In the liver of *db/db* mice, we detected a significant increase in the levels of 25-OHC, 27-OHC and 7α-OHCnone when compared to the db/lean mice (Fig. SI 4b). We also measured a decrease of about 40% of the 5α,6α-epoxycholesterol (Fig. SI 4b). Regarding the metabolism of the oxysterols measured here, there is no direct correlation between the mRNA expression levels of the enzyme involved in their metabolism and their levels. Indeed, the mRNA levels of cholesterol-25-hydroxylase (chol-25-OHase) and CYP7B1, responsible for the synthesis and catabolism, respectively, of 25-OHC, are both slightly increased (Fig. SI 4b).

**Figure 5:**
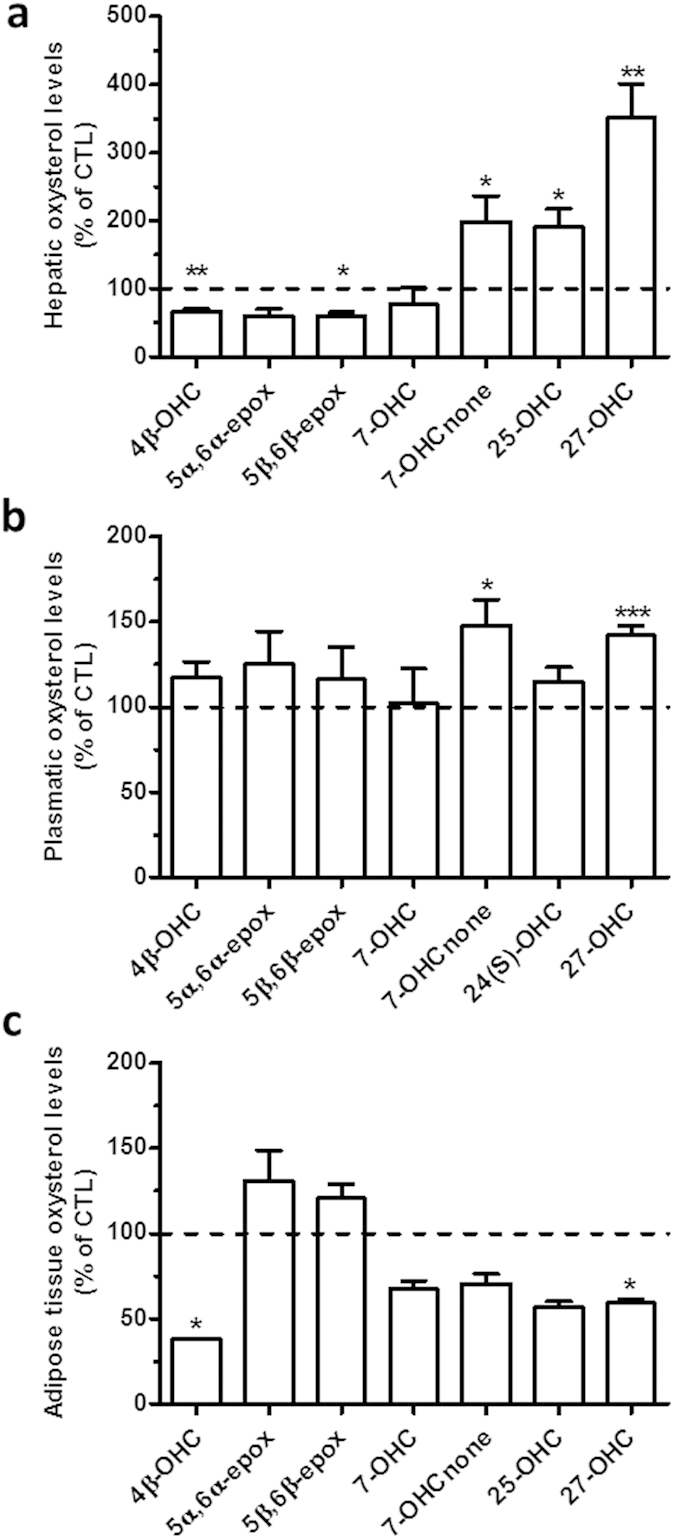
Oxysterol levels in the liver, plasma and adipose tissue of the *ob/ob* genetic model of obesity. Oxysterol levels were quantified by HPLC-MS in (**a**) the liver, (**b**) the plasma and (**c**) the adipose tissue in the *ob/ob* model. The data are reported as percentage of the control group (ob/lean) (shown as a dotted line). Data are mean ± s.e.m.; student’s t-test between *ob/ob* group and the ob/lean group *P < 0,05 and **P < 0,01 and ***P < 0,001.

Concerning the *ob/ob* mice, the levels of 4β-OHC are decreased in the liver of obese mice compared to their lean littermates (Fig. 5a) similarly to what was found in the diet-induced obesity model. Conversely, and as observed with the *db/db* mice, the levels of 25-OHC and 27-OHC are increased in the *ob/ob* mice compared to their lean control (ob/lean) (Fig. 5a). Although the expression of chol-25-OHase was not increased, part of the increased levels of 25-OHC could be explained by the drastic decrease in CYP7B1 mRNA expression (Fig. SI5). Another similarity between the two genetic models of obesity can be found in the decreased levels of 5α,6α-epoxycholesterol (Fig. 5a), which is one of the main ROS-derived cholesterol metabolites^43^. Regarding OHC plasmatic levels in the *ob*/*ob* model, as observed in the diet-induced obesity model, we measured an increase in the levels of 7-OHCnone and 27-OHC (Fig. 5b). Finally, the OHC profile in the adipose tissue of the *ob*/*ob* model displays a significant decrease in 4β-OH and 27-OHC levels (Fig. 5c) further supporting the notion that the adipose tissue does not act as storage compartment for these oxysterols.

### Primary bile acid levels are altered during obesity

**Figure 6:**
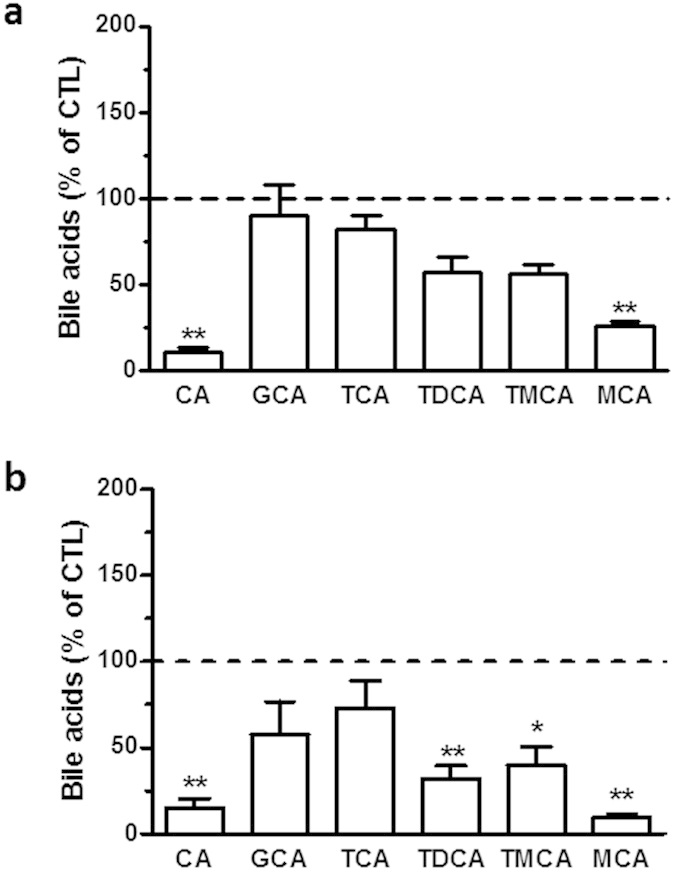
Obesity alters the hepatic levels of bile acids. Cholic acid (CA), glycocholic acid (GCA), taurocholic acid (TCA), taurodeoxycholic acid (TDCA), tauromuricholic acid (TMCA) and muricholic acid (MCA) levels were quantified by HPLC-MS in the liver of (**a**) the HFD mice at week 16 and their respective control group and (**b**) the *ob/ob* mice and their respective control. The data are reported as percentage of the respective control group (shown as a dotted line). Data are mean ± s.e.m.; student’s t-test between groups *P < 0,05 and **P < 0,01.

Oxysterols are bioactive lipids belonging to an important metabolic pathway with bile acids as downstream metabolites. Thus, we compared the levels of hepatic bile acids in the two genetic models of obesity and at week 16 of the diet-induced obesity model. The levels of cholic acid (CA) and muricholic acid (MCA) were decreased both in mice fed a high-fat diet and in *ob/ob* mice when compared to their respective control groups (Fig. 6a,b). We also observed a significant decrease of taurodeoxycholic acid (TDCA) and tauromuricholic acid (TMCA) in the liver of the *ob/ob* mice (Fig. 6b). However, there is no significant variation in bile acids levels in the *db/db* mice model (Fig. SI 4c).

Finally, to have a more complete picture of the changes induced by the high-fat feeding on the bile acid metabolism, we also measured the primary bile acid levels in the gallbladder of control and high-fat fed mice (at week 16). We found similar variations than those observed in the liver (Fig SI 6).

## Discussion

In our study, we sought to investigate the levels of key lipid mediators, namely the oxysterols, in models of obesity, either diet-induced or from genetic origins. Therefore we characterized the most abundant oxysterols in the liver, hypothalamus and adipose tissue, three organs greatly affected during metabolic syndrome, as well as in the plasma.

Although cytochromes are the key enzymes in the metabolism of oxysterols, we did not find any correlation between the changes in expression of these enzymes and the variation in oxysterols levels. To date, the biochemical tool of choice to study CYP enzymes in tissues is the study of their mRNA expression through RT-qPCR^44^,^45^. As mentioned, we could not observe a clear relation between oxysterol levels and enzymes mRNA expression levels. This observation can be partly explained by the complexity of oxysterol metabolic pathways. Indeed, a given cytochrome P450 has multiple oxysterols as substrates, and a given oxysterol can be metabolized by several cytochromes (Figs 1 and 2).

The question remains on how 4β-OHC and 7-OHC can be present in the hypothalamus since the main enzymes responsible for their production in mice (CYAP3A11 and CYP7A1, respectively) are not expressed in this tissue. Several hypotheses, not mutually exclusive, could explain this phenomenon. As oxysterol metabolism in mice is less characterized than in humans, it is possible that other cytochromes (or enzymes) than the CYAP3A11 and CYP7A1 could synthetize 4β-OHC and 7-OHC. Another possibility is the crossing of the blood-brain barrier by the oxysterols. For instance it was shown that 27-OHC is able to enter the brain from the periphery^46^. Another possibility, at least for 4β-OHC, is that it comes from another part of the CNS where CYP3A11 is expressed. For instance, we found expression of the CYP3A11 in the hindbrain of these mice. Finally, it is also well established that beside enzymatic formation, ring substituted oxysterols can be endogenously formed through ROS oxidation^14^.

Cholesterol is prone to oxidation and thus increased tissue cholesterol levels could result in higher levels of non-enzymatically produced oxysterols. Here we found increased hepatic cholesterol levels in the three models of obesity (Fig. SI 1h–j). Interestingly however, 5α,6α-epoxycholesterol and 5β,6β-epoxycholesterol, i.e. two major products of non-enzymatic cholesterol oxidation, were not increased in obese animals. Furthermore, we did not find any correlation between the hepatic levels of cholesterol and oxysterols.

Because the diet could be a source of oxysterols, we compared the levels of oxysterols in the normal and high-fat diets. We found higher levels of 4β-OHC, 25-OHC and 27-OHC in the high-fat diet, whereas the levels of 7-OHC, 5α,6α-epoxycholesterol and 5β,6β-epoxycholesterol were similar to those measured in the normal diet (Fig. SI 7). However the changes found in the plasma after 1 week of high-fat diet did not reflect the differences in oxysterol content of the diets. For instance 27-OHC is more abundant in the high-fat diet when compared to the normal diet (Fig. SI 7), but it is not increased in the plasma of the diet-induced obesity mice after 1 week of diet. Thus, it is unlikely that the oxysterols present in the diets directly contributed to the variations of oxysterol levels detected in the tissues. Taken together, these elements indicate that the variations in oxysterol levels that we found in obesity are mainly due to changes in oxysterols’ metabolic pathways.

As we found marked alterations in hepatic oxysterol levels in the three models of obesity, we performed a hierarchical clustering analysis to investigate whether we could distinguish between lean and obese subjects. We chose to use the three oxysterols that showed the more marked variations, namely 4β-OHC, 27-OHC and 7α-OHCnone as well as the three main enzymes involved in their respective synthesis, CYP3A11, CYP27A1 and CYP7A1. This analysis associates the two mice showing the most similarities into a same cluster. Then this new formed cluster is merged with the closest cluster (most similar cluster). Thus, the dendrogram obtained displays the hierarchical ordering of data or mice. Even though the three models of obesity used showed differences regarding oxysterol levels and oxysterol metabolism, this statistical analysis enabled us to discriminate, almost perfectly, between a “lean” phenotype (comprised of the control mice of the high-fat diet study at week 16, the db/lean mice and the ob/lean mice) and an “obese” phenotype (comprised of the high-fat diet mice at week 16, the *db/db* mice and the *ob/ob* mice) (Fig. SI 8).

Oxysterols are further transformed, through CYP 450 action, into primary bile acid which are more hydrophilic and are destined to be secreted by hepatocytes hence forming part of the biliary secretion. In the *ob/ob* model, we found decreased levels of cholic acid (CA) and taurodeoxycholic acid (TDCA), two bile acids derived from the classic pathway, as well as a decrease of muricholic acid (MCA) and tauromuricholic acid (TMCA), two bile acids resulting from the alternative pathway, in the liver of the obese mice. We also found a decreased mRNA expression of CYP7A1 and CYP27A1, the rate limiting enzyme of the classic pathway and the first enzyme of the alternative pathway of bile acid synthesis, respectively^47^,^48^. Concerning the high-fat diet mice at week 16, because they show an increase in CYP7A1 expression, we would expect an increase in bile acids derived from the classic pathway. However we found decreased hepatic levels of CA and TDCA. We, therefore, hypothesized that a down-regulation of the expression of CYP8B1, an enzyme downstream of CYP7A1, could be involved. And, surprisingly, we found that the expression of CYP8B1 is increased (3,1-fold increase; p-value of 0,0033). This could suggest that bile acid levels are decreased in the liver, even though their synthesis seems to be increased, because their hepatobiliary excretion is increased^49^. To assess this hypothesis, we measured the bile acid levels in the gallbladder of the diet-induced obesity model at week 16 (Fig. SI6). We found the same trends in bile acid variations in both liver and gallbladder (decreased levels of CA and MCA). Therefore, with the increased 7α-OHCnone hepatic levels, increased CYP8B1 expression and decreased levels of bile acids in both liver and gallbladder, a remaining possibility could be a decreased reabsorption of bile acids in diet-induced obesity in mice.

Finally, from a translational point of view, it is interesting to mention that it was also recently found that 4β-OHC plasma levels were decreased in human obese patients^50^. Thus this decrease in 4β-OHC seems to be a general feature of obesity. Interestingly, this is inversely associated with the content of 4β-OHC in the diet of mice, thereby reinforcing the involvement of endogenous metabolic pathways. However, the precise role(s) and putative mechanism(s) of action of 4β-OHC remain to be elucidated. Among the possible mechanisms, oxysterols are ligands of the nuclear receptors LXR α and β. These receptors play a central role in the cholesterol homeostasis by modulating the expression of genes involved in cholesterol absorption, uptake or biosynthesis^51^. LXRs are also known as modulators of inflammatory tone. Indeed, upon activation, they have been shown to repress the expression of several pro-inflammatory genes^52^,^53^. However, the various effects of oxysterols are not only restricted to LXRs activation. Indeed, 27-OHC has been recently identified as an endogenous selective estrogen receptor modulator (SERM). Oxysterols can also act through Epstein-Barr virus induced gene 2 (aka EBI2 or GPR183) and modulate inflammation notably in viral infection^54^,^55^ or act through other nuclear receptors, such as the retinoic acid receptor-related orphan receptors (RORs)^56^,^57^. The complexity and multiplicity of the pathways mediating the effects of oxysterols are, of course, difficult to unravel. Therefore, it is crucial to first characterize the profile of oxysterol species in a specific pathophysiological setting.

Taken together, our findings show an altered oxysterol metabolism in obesity settings. Interestingly, these alterations were partly different depending on the model of obesity considered. Nevertheless, one of the most striking findings of this work is the marked and persistent decrease of 4β-OHC hepatic hypothalamic, plasmatic and adipose tissue levels throughout the high-fat diet time course study. The same decrease was also found in the liver and adipose tissue of the *ob/ob* mice. As the properties and physiopathological relevance of this oxysterol remain poorly documented further studies are warranted to fully unravel the potential of 4β-OHC, and of other oxysterols, during obesity.

## Materials and Methods

### Animals and diets

High-fat diet model: Nine weeks old male C57BL/6 mice (Charles River) were housed in a controlled environment (12-h day light cycle, lights-off at 6 pm, controlled temperature and humidity). Upon arrival, they were split into twelve groups of eight mice each (4 mice/cage) and acclimated for one week. Then, six of these groups were given free access to a standard diet (AIN 93-M, Research Diets, New Brunswick, USA) and the remaining six groups were given free access to a high-fat diet (D12492, Research Diets, New Brunswick, USA). Of note, cholesterol content in the standard and high-fat diet was 28mg/kg and 280 mg/kg, respectively. The percentage of kcal derived from fat, carbohydrate and protein as well as the fatty acid composition of both diets are reported in Table S5 (according to the manufacturer). For this experiment, one group under standard diet and one group under high-fat diet were euthanized at each selected time-point (i.e. after 1, 2, 4, 6, 8 and 16 weeks). Three hours prior to the sacrifice, mice body composition in terms of fat and lean masses was determined using a 7,5-MHz time-domain NMR (LF50 Minispec; Bruker, Rheinstetten, Germany).

Genetic models of obesity were also used in this study. Thus, obese (B6.V-Lepob/J mice) and diabetic (BKS.Cg-m+/+ Leprdb/J) mice (from the Jackson Laboratory) and their respective lean controls were housed in the same controlled environment and given free access to the standard diet (A04, Villemoisson sur Orge, France).

Mice were anesthetized using isoflurane after a 6h fasting period and blood collected from the portal vein. Mice were then sacrificed by cervical dislocation. Tissues were collected, snap-frozen in liquid nitrogen and stored at −80 °C until further analysis.

The study was performed in accordance with the European recommendation 2007/526/CE (which was transformed into the Belgian Law of May 29, 2013), regarding the protection of laboratory animals. The animal experimentation ethics committee from the Université catholique de Louvain approved the protocol of the study (study agreement 2010/UCL/MD/022; lab agreement LA1230314).

### RNA preparation and RT-qPCR analysis

Total RNA from tissues was extracted using TriPure® reagent (Roche, Basel, Switzerland) according to the manufacturer’s instructions. cDNA was synthesized using an RT kit (Promega, GoScript™ Reverse Transcription System) from 1 μg of total RNA. qPCR was performed with a StepOnePlus instrument and software (Applied Biosystems, Foster City, CA, USA). PCR reactions were run using a SYBR Green mix (Promega, GoTaq® qPCR Master Mix). Each sample was measured in duplicate during the same run. The following conditions were used for amplification: an initial holding stage of 10 min at 95 °C, then 45 cycles consisting of denaturation at 95 °C for 3 s, annealing at 60 °C for 26 s, and extension at 72 °C for 10 s. Products were analyzed by performing a melting curve at the end of the PCR reaction. Data are normalized to the 60S ribosomal protein L19 (RPL19) mRNA expression^58^. The sequences of the primers used are listed in table 1. All the primers used are intron-skipping except for CYP8B1 and Chol25OHase as there is only one coding exon.

### Oxysterols quantification

We used a HPLC-MS method we recently developed and validated that allows for the quantification of the endogenous oxysterols while avoiding the artefactual formation of oxysterols from cholesterol^31^. Of note, for the hypothalamus, oxysterol quantification was performed on the same samples used for RT-qPCR. Following the RNA extraction, the remaining chloroform fraction was added into a glass vial containing dichloromethane-methanol-bi-distilled water (4:2:1; v/v/v). d_7_-4β-hydroxycholesterol (133.3 pmol) and d_7_-24-hydroxycholesterol (200 pmol) were added as internal standards. Tissues were directly homogenized in a glass vial containing 8 mL dichloromethane. Internal standards were then added. The same proportion (4:2:1; v/v/v) in the extraction mixture was achieved by adding methanol and bi-distilled water to the vials. For all tissues, 10 μg of butylated hydroxytoluene (BHT) and 20 ng of EDTA were added to the mixture to avoid artefact generation due to cholesterol oxidation during the procedure. Vials were then shaken vigorously and sonicated. After centrifugation, the organic phase was recovered and evaporated under a nitrogen stream to minimize the risk of oxidation. Oxysterols were pre-purified by solid-phase extraction (SPE). Cholesterol was eliminated using hexane-isopropanol, after which the oxysterols were eluted by increasing the proportion of isopropanol. The oxysterol fraction was analyzed by HPLC-MS using a LTQ-Orbitrap mass spectrometer (ThermoFisher Scientific) coupled to an Accela HPLC system (ThermoFisher Scientific). Analyte separation was achieved using a C-18 Supelguard pre-column and a kinetex LC-18 column (5µm, 4.6 × 150 mm) (Phenomenex). Mobile phases A and B were composed of MeOH-H_2_O-acetic acid 75:25:0.1 (v/v/v) and MeOH-acetic acid 100:0.1 (v/v), respectively. The gradient (0.4 mL/min) was designed as follows: transition from 100% A to 100% B linearly over 15 min, followed by 10 min at 100% B and subsequent re-equilibration at 100% A. We performed MS analysis in the positive mode with an APCI ionization source. The oxysterols were quantified by isotope dilution using d_7_-24-hydroxycholesterol for those oxidized on the lateral chain and d_7_-4β-hydroxycholesterol for those oxidized on the sterol backbone. The data were normalized by tissue samples weight.

### Bile acids quantification

To measure bile acid content, tissue samples were homogenized in 600 μl of ice-cold distilled water and proteins precipitated using acetone. Concerning the gallbladder, 5μl of its content were homogenized in 100 μl of ice-cold distilled water and the proteins precipitated using acetone. The samples were next centrifuged and the supernatant evaporated to dryness. The resulting residue was analyzed by HPLC-MS using a LTQ-Orbitrap mass spectrometer (ThermoFisher Scientific) coupled to an Accela HPLC system (ThermoFisher Scientific). Analyte separation was performed on a C-18 Supelguard pre-column and a kinetex LC-18 column (5µm, 4.6 × 150 mm) (Phenomenex). Mobile phases A and B were composed of MeOH-H_2_O-ammonium hydroxide 50:50:0.1 (v/v/v) and MeOH-ammonium hydroxide 100:0.1 (v/v) respectively. The gradient (0.4 mL/min) was as follows: transition from 100% A to 60% and 40% A and B linearly over 15 min. B increases then gradually to achieve 100% 4 min later and kept at that level for and an additional 10 min before re-equilibration at 100% A. We performed MS analysis in the negative mode with an ESI ionization source. Data are expressed as percentage of control condition and were normalized by tissue samples weight.

### Glucose, triglycerides and total cholesterol content

Plasma glucose, total cholesterol and triglycerides were quantified using an enzymatic reaction coupled with a spectrophotometric detection of the end-product (DiaSys Diagnostic and Systems, Holzheim, Germany). The same kits were used to quantify the total cholesterol content of the liver and subcutaneous adipose tissue as well as the triglycerides content of the liver after a chloroform-methanol lipid extraction^59^.

### Histology

Paraffin-embedded mouse liver sections of 2μm were deparaffinized in toluene and then rehydrated in baths of decreasing ethanol content. Coloration was performed using Mayer’s hematoxylin and eosin. Slides were mounted with DPX mounting media (Merck Millipore) and scanned with a Leica SCN400 Slide Scanner. The images obtained were analyzed using ImageJ software (http://imagej.nih.gov/ij/).

### Statistical analysis

For the high-fat diet study, at each selected time-points, the high-fat diet group was compared to its respective control (CTL) group. Data are presented as mean ± SEM. Statistical analysis were performed using GraphPad Prism version 5.0 for Windows (San Diego, CA). We used the two tailed Student’s t-test for unpaired values to compare two groups and when relevant we used the Mann-Whitney test. Statistical significance was taken when p < 0.05. The hierarchical cluster analysis using Ward method was performed using JMP®, Version 11 (SAS Software). This analysis was conducted on the control and HFD mice of the diet-induced obesity model at week 16 and on the mice of the two genetic models of obesity.

## Additional Information

**How to cite this article**: Guillemot-Legris, O. *et al.* Obesity is associated with changes in oxysterol metabolism and levels in mice liver, hypothalamus, adipose tissue and plasma. *Sci. Rep.* **6**, 19694; doi: 10.1038/srep19694 (2016).

## Supplementary Material

Supplementary Information

## Figures and Tables

**Table 1:** Primer sequences.

Gene	Forward Primer (5′-3′)	Reverse Primer (5′-3′)
Chol25OHase	CTGACCTTCTTCGACGTGCT	GGGAAGTCATAGCCCGAGTG
CYP3A11	GAAGCATTGAGGAGGATCACA	GTGCCTAAAAATGGCAGAGG
CYP7A1	GGGATTGCTGTGGTAGTGAGCTG	GGTATGGAATCAACCCGTTGTC
CYP7B1	TAGGCATGACGATCCTGAAA	TCTCTGGTGAAGTGGACTGAAA
CYP27A1	GGCTACCTGCACTTCCT	CTGGATCTCTGGGCTCTTTG
CYP39A1	TGGTGGAGACGGAAACAC	AGAATGGAGACAACATCAGCA
CYP46A1	ATGAGGTTGTCGGTTCCAAG	AGGTGCTGAACAGGAGAGGA
CYP8B1	GATCCGTCGCGGAGATAAGG	CGGGTTGAGGAACCGATCAT
F4/80	TGACAACCAGACGGCTTGTG	CAGGCGAGGAAAAGATAG
GFAP	TTCGCACTCAATACGAGGCA	CTCCAGATCGCAGGTCAAG
IL-6	ACAAGTCGGAGGCTTAATTACACAT	TTGCCATTGCACAACTCTTTTC
RPL 19	TGACCTGGATGAGAAGGATGAG	CTGTGATACATATGGCGGTCAATC
Serpina3n	GGACATTGATGGTGCTGGTGAAT	CTCCTCTTGCCCGCGTAGAA
SULT2B1b	GCTCCAAGGCTAAGGTGATTT	TGAAGGAACTGGTCGGGTGT
TLR4	TGCAGAAAATGCCAGGATGATG	AACTACCTCTATGCAGGGATTCAAG
