# Supplementary material for: Obesity is associated with changes in oxysterol metabolism and levels in mice liver, hypothalamus, adipose tissue and plasma
Source: Sci Rep. 2016 Jan 22;6:19694. doi: 10.1038/srep19694 (PMC4726335; doi:10.1038/srep19694)
Supplement: Supplementary Information [file srep19694-s1.pdf]

Supplementary information

Obesity is associated with changes in oxysterol metabolism and levels in mice liver, hypothalamus, adipose tissue and plasma

Owein Guillemot-Legris[1#], Valentin Mutemberezi[1#], Patrice D. Cani[2], Giulio G. Muccioli[1]*

SI 1.

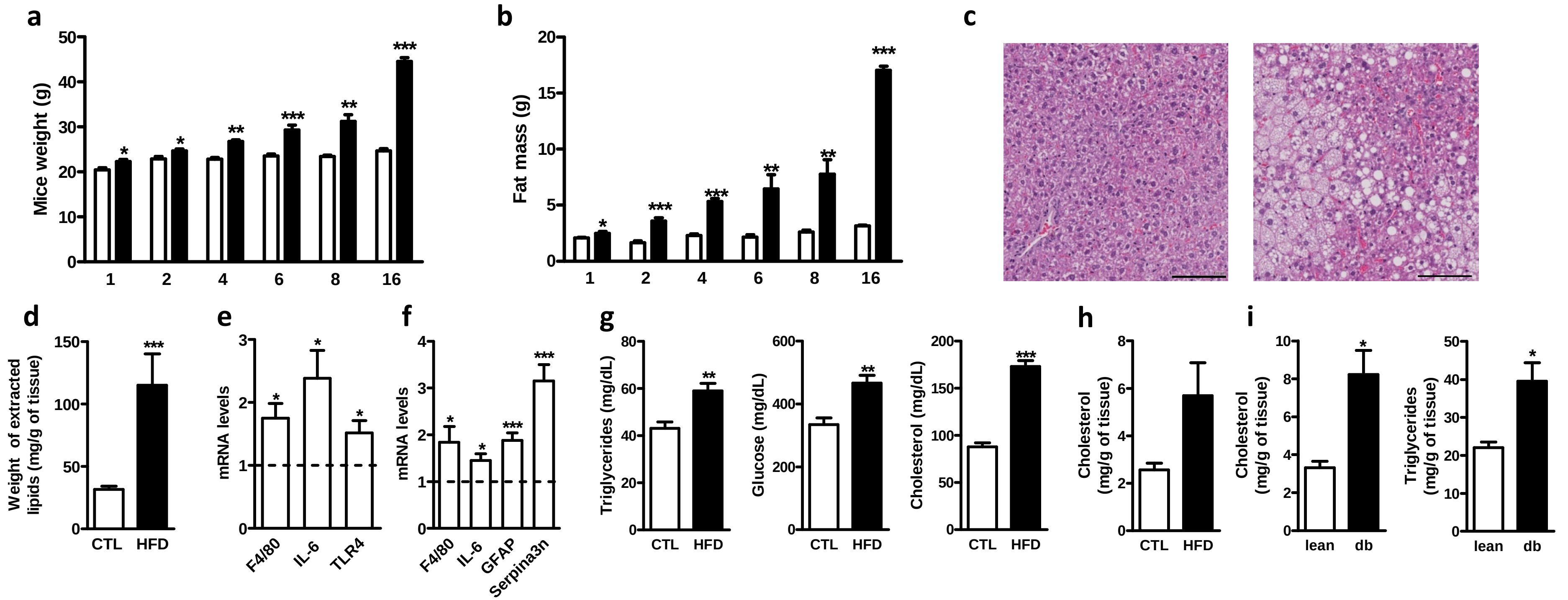

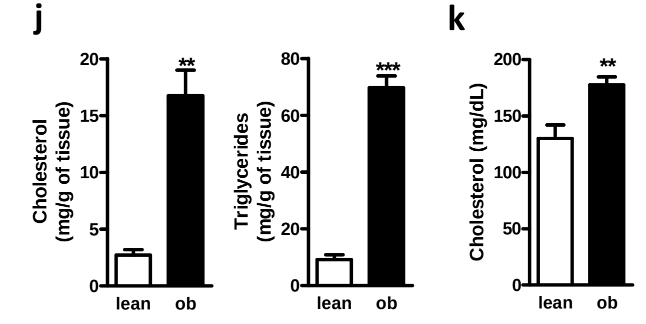

SI 1. Assessment of the obese phenotype and the subsequent metabolic inflammation.
(**a**) Weight of the mice of the CTL group (white column) and HFD group (black column) at each selected time point, the day of the sacrifice. (**b**) Fat mass of the mice measured by time domain NMR of the CTL group (white column) and HFD group (black column) at each selected time point, the day of the sacrifice. (**c**) Representative photomicrographs of (left) a control and (right) HFD livers of mice at week 16 stained with HE (bar=100µm). (**d**) Weight of the total extracted lipids in the liver of CTL and HFD mice at week 16. (**e**) mRNA expression of F4/80, a myeloid lineage marker, IL-6 and TLR4 in the liver of CTL and HFD mice at 16 weeks. (**f**) mRNA expression of F4/80, IL-6, GFAP and Serpina3n in the hypothalamus of CTL and HFD mice at 16 weeks. (**g**) Plasmatic levels of triglycerides, glucose and cholesterol of CTL and HFD mice at week 16. (**h**) Hepatic levels of cholesterol of CTL and HFD mice at week 16. (**i**) Hepatic levels of cholesterol and triglycerides of lean and *db/db* mice. (**j**) Hepatic levels of cholesterol and triglycerides of lean and *ob/ob* mice. (**k**) Plasmatic levels of cholesterol of lean and *ob/ob* mice.
For **e**-**f**, data are expressed relative to the control group set at 1 (dotted line).
Data are mean ± s.e.m.; student's t-test between HFD group and its CTL group or *db/db* vs db/lean or *ob/ob* vs ob/lean *P<0,05 ; **P<0,01 and ***P<0,001.

SI 2.

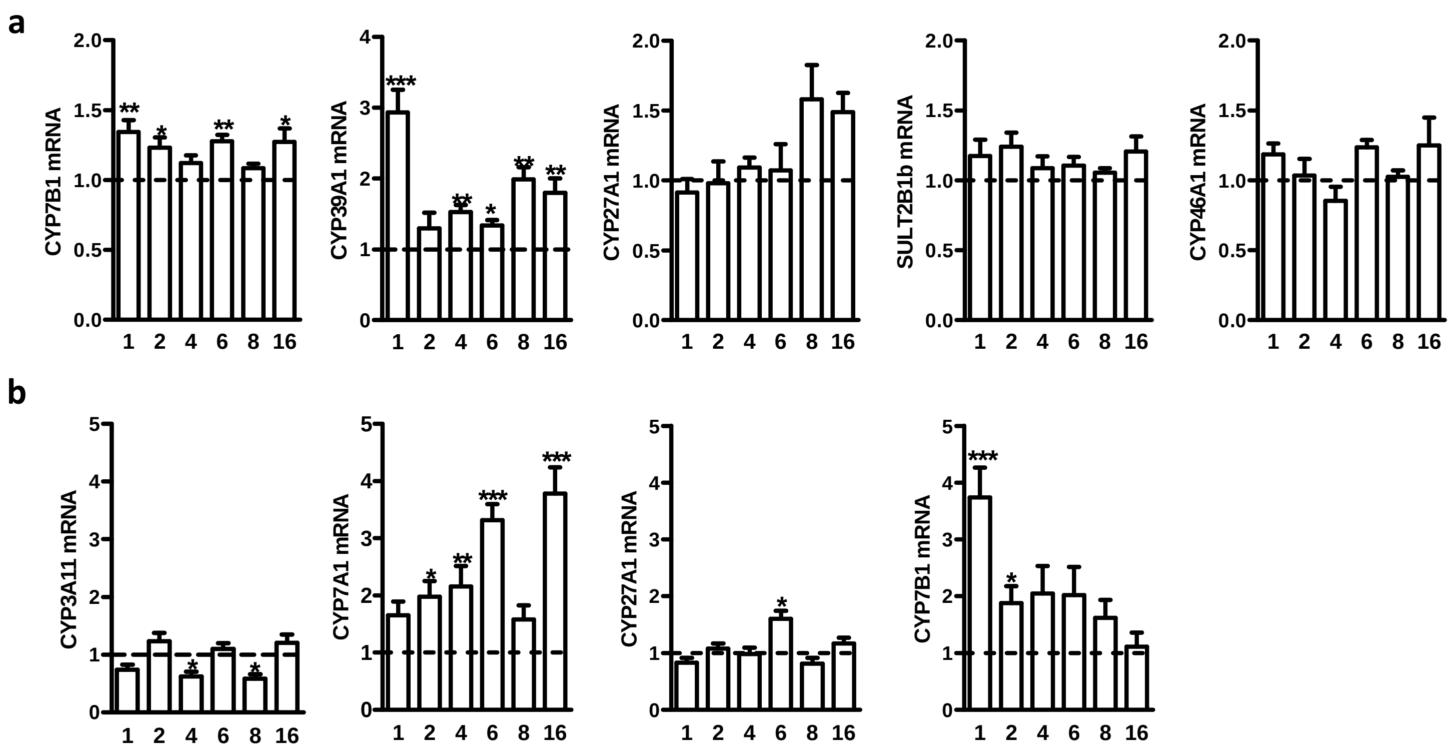

SI 2. **Hypothalamic and hepatic expression of the oxysterol metabolizing enzymes during the development of diet-induced obesity.** Relative mRNA expression levels of the enzymes metabolizing oxysterols at the different time-points throughout the diet-induced obesity study. At each time point (i.e. 1; 2; 4; 6; 8 and 16 weeks) a control and a high-fat group were sacrificed. Enzyme expression was measured by qRT-PCR in (**a**) the hypothalamus and (**b**) the liver. The data are reported relative to the control group set at 1 (shown as a dotted line). Data are mean ± s.e.m.; student's t-test between HFD group and its control group *P<0,05; **P<0,01 and ***P<0,001

SI 3.

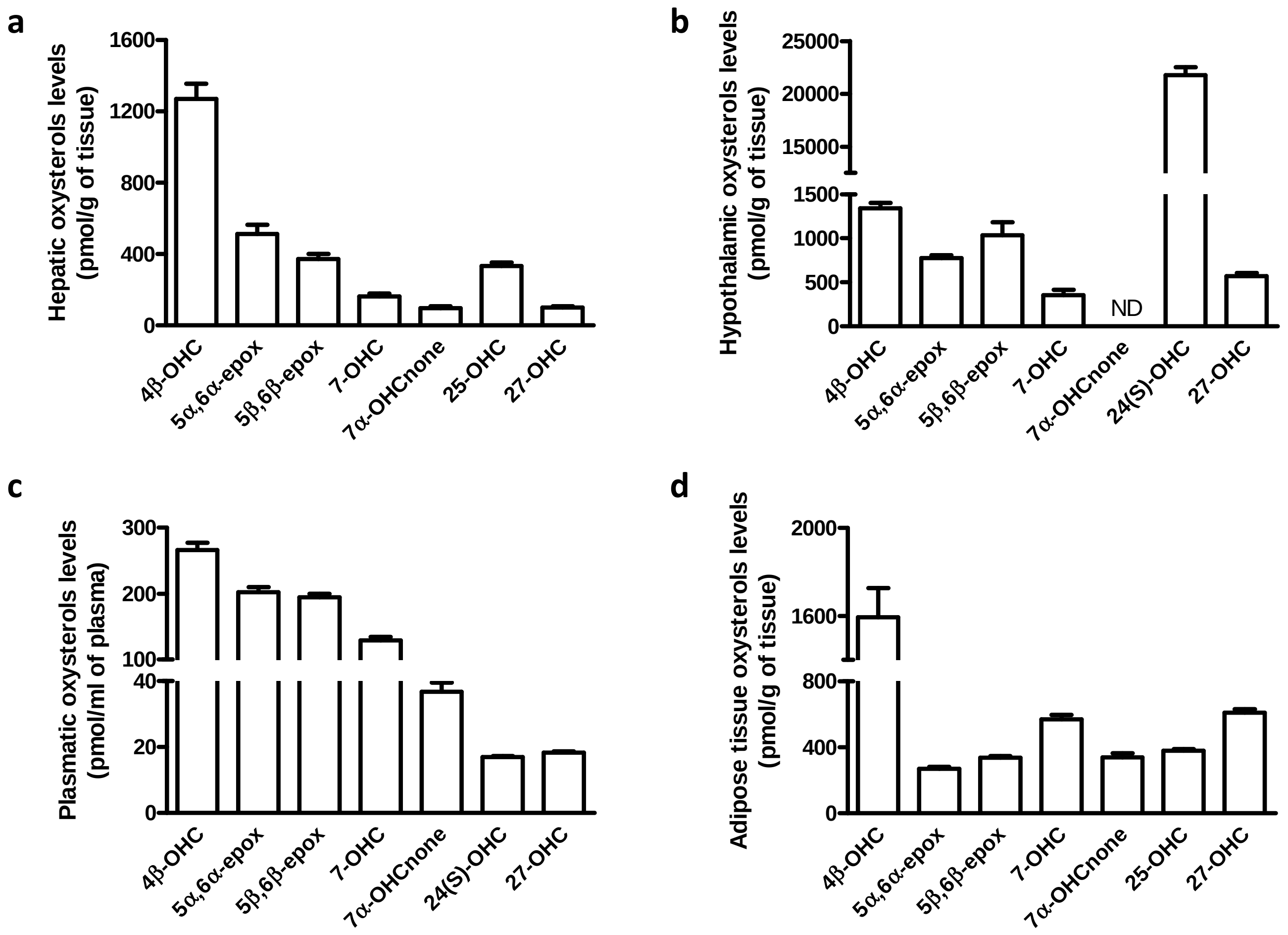

SI 3. Absolute levels of oxysterol in the liver, hypothalamus, plasma and adipose tissue of C57BL/6 mice under control diet. Oxysterol levels measured in the (**a**) liver, (**b**) hypothalamus, (**c**) plasma and (**d**) adipose tissue of the control groups used in the HFD time course study. Levels are expressed as pmol/g of tissue or pmol/ml of plasma. Data are mean ± s.e.m.

SI 4.

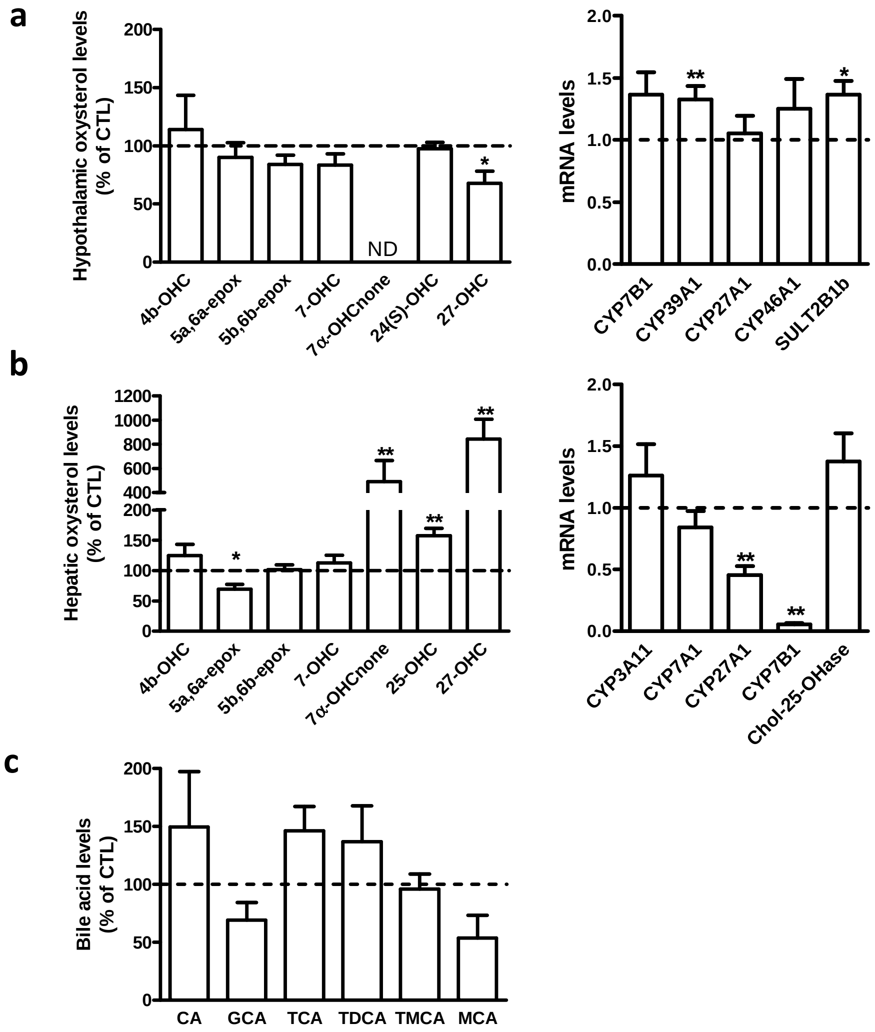

SI 4. Oxysterol metabolism in the *db/db* mice.
Oxysterol levels and mRNA expression of the enzymes metabolizing the oxysterols in (**a**) the hypothalamus and (**b**) the liver. The data are expressed relative to the db/lean control group (shown as a dotted line) set at 100 for lipid levels and at 1 for mRNA expression. (**c**) Hepatic bile acids level relative to control (db/lean) set at 100. Data are mean ± s.e.m.; student's t-test between *db/db* group and its control group *P<0,05 and **P<0,01

SI 5.

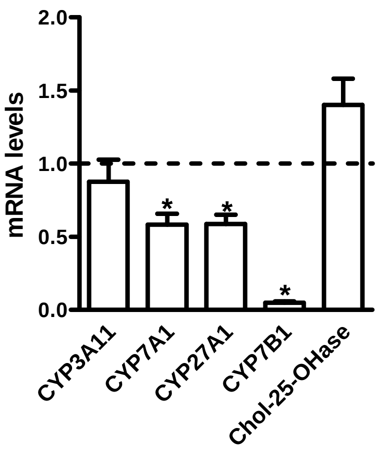

SI 5. Hepatic expression of the oxysterol metabolizing enzymes in the ob/ob mice
Relative mRNA expression levels of the enzymes metabolizing oxysterols. The data are reported relative to the control group (ob/lean) set at 1 (shown as a dotted line). Data are mean ± s.e.m.; student's t-test between *ob/ob* group and its control group *P<0,05

SI 6.

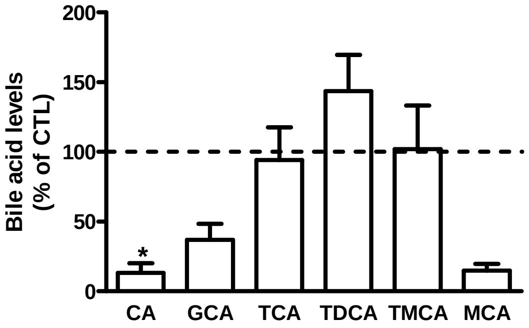

SI 6. Bile acids levels in the gallbladder in the diet induced obesity model at week 16. Bile acids level relative to control of the HFD mice at week 16 in the gallbladder. The CTL levels are set at 100. Data are mean ± s.e.m.; student's t-test between the HFD and normal chow group *P<0,05

SI 7.

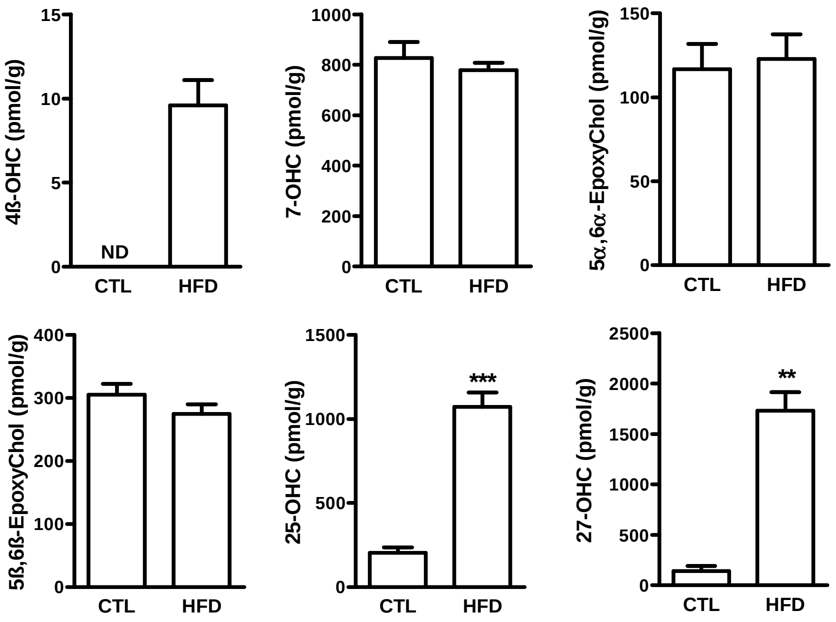

SI 7. Oxysterol levels in the control and high-fat diet of the diet-induced obesity model.
OHC levels measured in the standard diet (AIN93M, **CTL**) and in the high-fat diet (D12492, **HFD**) used in the time course diet-induced obesity study. Data are mean ± s.e.m.; student's t-test between HFD group and the CTL group **P<0,01 and ***P<0,001.

SI 8.

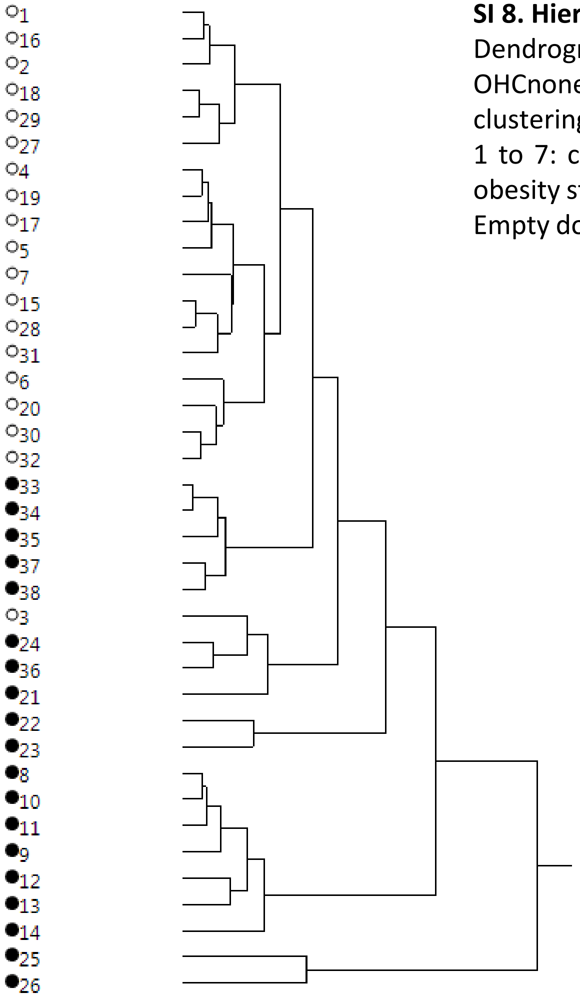

SI 8. Hierarchical clusterization of mice of the three obesity models used.

Dendrogram tree diagram representing the hierarchical clustering analysis in the liver of 4β-OHC, 27-OHC, 7α-OHCnone, CYP 3A11, CYP 27A1 and CYP 7A1 for our three models of obesity. This analysis demonstrates a clear clustering between a « lean » phenotype and an « obese » phenotype.

1 to 7: control mice from the diet-induced obesity study (week 16); 8 to 14: HFD mice from the diet-induced obesity study (week 16); 15 to 20: db/lean; 21 to 26: *db/db*; 27 to 32: ob/lean; 33 to 38: *ob/ob*.

Empty dots: lean mice ; Full dots: obese mice

SI 9.

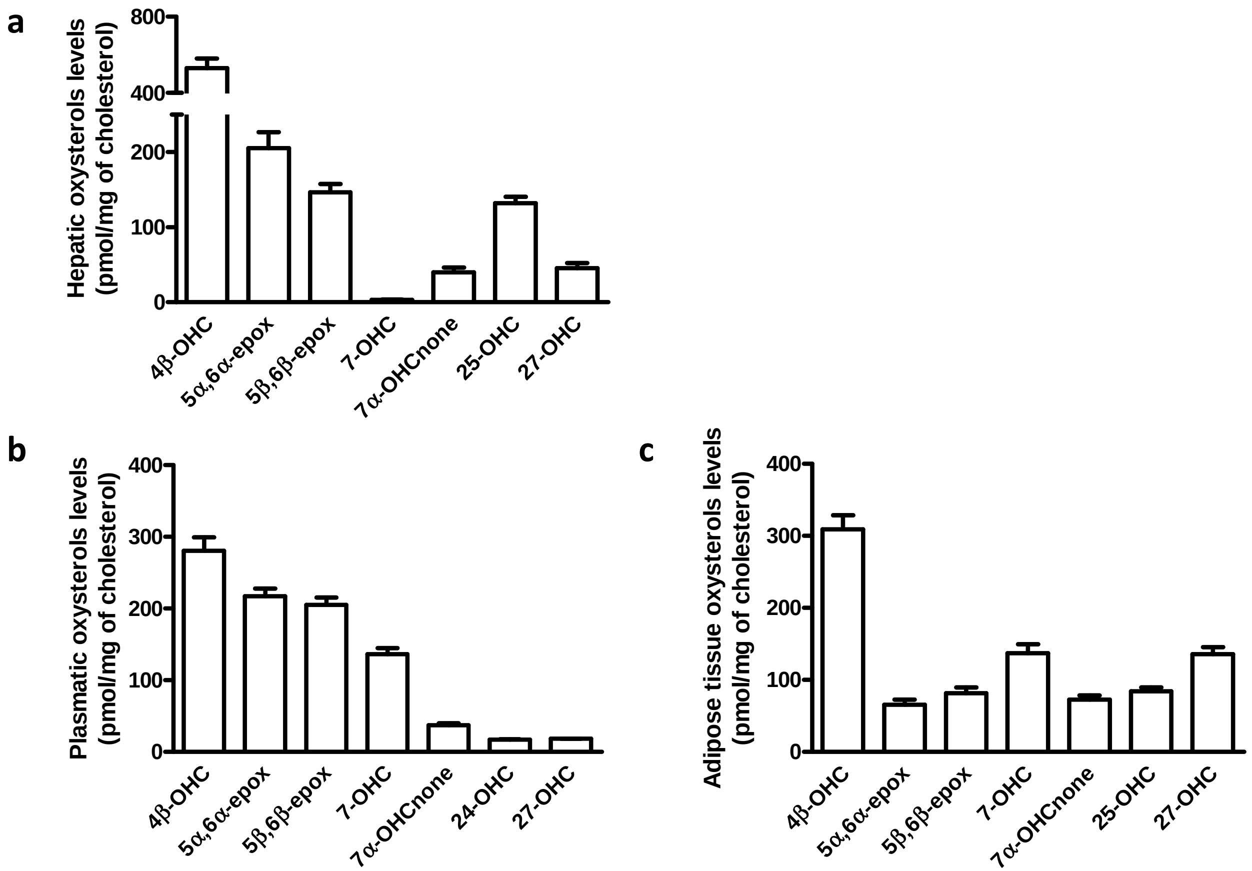

SI 9. Oxysterol levels in mice fed a standard diet normalized to cholesterol content in each tissue
Levels measured in the (**a**) liver, (**b**) plasma and (**c**) adipose tissue of the control groups used in the diet-induced obesity model. Data are mean ± s.e.m.

Table S1. Hypothalamic oxysterol levels are altered by the high-fat diet

Oxysterol levels (pmol/g of tissue) in the hypothalamus of the HFD group normalized to the levels found in the respective normal diet groups (**Fig. SI 3b**). Data are mean ± s.e.m. Student's t-test between HFD group and the respective CTL group **P<0,001.

HYPOTHALAMUS						
	**4β-OHC**	**5α,6α-epoxyChol**	**5β,6β-epoxyChol**	**7-OHC**	**24(*S*)-OHC**	**27-OHC**
**1 week**	82,8 ± 4,4	103,1 ± 9,5	101,8 ± 14,1	58,3 ± 5,2	109,3 ± 2,2	91,7 ± 5,2
**2 weeks**	70,9 ± 12,7	51,6 ± 8,9	68,8 ± 11,8	49,7 ± 5,9	80,0 ± 10,5	80,6 ± 12,5
**4 weeks**	89,2 ± 7,6	119,1 ± 5,8	148,2 ± 13,0	116,4 ± 23,8	97,4 ± 1,4	107,6 ± 11,3
**6 weeks**	73,9 ± 2,0 **	95,2 ± 3,0	95,6 ± 6,3	95,0 ± 8,1	100,9 ± 1,4	99,4 ± 5,7
**8 weeks**	63,6 ± 3,3 **	84,4 ± 7,0	111,7 ± 16,5	134,8 ± 24,6	100,8 ± 2,1	87,3 ± 5,1
**16 weeks**	37,3 ± 3,2 **	103,3 ± 9,2	130,8 ± 25,5	86,4 ± 17,3	82,8 ± 5,7	90,4 ± 4,7

Table S2. Hepatic oxysterol levels are altered by the high-fat diet

Oxysterol levels (pmol/g of tissue) in the liver of the HFD group normalized to the levels found in the respective normal diet groups (**Fig. SI 3a**). Data are mean ± s.e.m. Student's t-test between HFD group and the respective CTL group *P<0,01 and **P<0,001.

LIVER							
	**4β-OHC**	**5α,6α-epoxyChol**	**5β,6β-epoxyChol**	**7-OHC**	**7-OHCnone**	**25-OHC**	**27-OHC**
**1 week**	62,7 ± 4,2 **	122,0 ± 7,1	113,3 ± 5,8	127,1 ± 23,7	112,1 ± 15,5	69,0 ± 2,5	73,5 ± 3,6
**2 weeks**	67,9 ± 4,0 *	105,2 ± 6,6	129,4 ± 14,0	116,5 ± 11,9	181,1 ± 24,7 *	90,3 ± 3,5	81,3 ± 14,9
**4 weeks**	62,3 ± 4,5 **	189,9 ± 62,1	144,6 ± 40,7	88,7 ± 9,7	143,4 ± 23,3	80,6 ± 4,0	71,3 ± 7,7
**6 weeks**	60,8 ± 5,8 **	82,6 ± 8,9	91,2 ± 6,3	134,8 ± 31,5	186,9 ± 18,5	81,6 ± 4,9	128,8 ± 12,2
**8 weeks**	48,1 ± 2,5 **	87,1 ± 7,4	96,4 ± 8,5	112,5 ± 12,5	200,4 ± 19,5 *	86,5 ± 3,6	138,0 ± 19,3
**16 weeks**	32,1 ± 1,4 **	71,1 ± 14,1	108,4 ± 24,1	97,8 ± 14,0	275,0 ± 14,6 **	96,7 ± 5,7	156,6 ± 11,6 *

Table S3. Oxysterol levels, normalized to the cholesterol content, and relative to respective CTL

Oxysterol levels (pmol/mg of cholesterol) in the liver, adipose tissue and plasma of the HFD group normalized to the levels found in the respective normal diet group (**Fig. SI 9**). Data are mean ± s.e.m.; student's t-test between HFD group and the respective CTL group *P<0,01 and **P<0,001.

**LIVER**							
	**4β-OHC**	**5α,6α-epoxyChol**	**5β,6β-epoxyChol**	**7-OHC**	**7-OHCnone**	**25-OHC**	**27-OHC**
**1 week**	75,1 ± 3,8	149,4 ± 9,4 *	140,7 ± 7,6 *	158,8 ± 27,2	127,5 ± 18,7	83,3 ± 2,9	86,8 ± 3,5
**2 weeks**	71,5 ± 5,2	103,7 ± 3,6	121,7 ± 7,1	123,1 ± 11,1	195,9 ± 25,4 *	95,7 ± 2,9	88,5 ± 18,2
**4 weeks**	59,9 ± 4,8 **	72,7 ± 14,7	102,4 ± 21,8	86,7 ± 9,2	136,7 ± 26,3	79,2 ± 7,7	68,9 ± 7,1
**6 weeks**	57,7 ± 9,1	90,0 ± 14,0	80,4 ± 10,6	104,3 ± 19,1	193,7 ± 23,7	78,0 ± 12,2	102,2 ± 3,1
**8 weeks**	38,5 ± 3,6 **	70,5 ± 8,3	91,0 ± 12,5	92,7 ± 15,1	174,1 ± 19,2	76,0 ± 6,7	113,8 ± 20,0
**16 weeks**	17,5 ± 4,1 *	52,6 ± 15,8	61,2 ± 17,7	54,2 ± 16,4	146,8 ± 26,1	54,9 ± 14,1	109,4 ± 27,0
**ADIPOSE TISSUE**							
	**4β-OHC**	**5α,6α-epoxyChol**	**5β,6β-epoxyChol**	**7-OHC**	**7-OHCnone**	**25-OHC**	**27-OHC**
**1 week**	29,2 ± 5,0	59,8 ± 8,7	68,3 ± 12,1	57,6 ± 5,7	111,8 ± 19,5	52,4 ± 8,4	56,9 ± 11,2
**2 weeks**	30,0 ± 2,8 **	35,6 ± 2,1 **	34,6 ± 1,7	29,5 ± 2,9 **	141,5 ± 14,0	45,1 ± 4,3 **	38,3 ± 3,6 **
**4 weeks**	24,3 ± 2,6 **	26,8 ± 3,1 *	31,0 ± 2,9	26,2 ± 1,5	98,9 ± 6,8	53,7 ± 2,4 **	43,8 ± 1,8 **
**6 weeks**	26,2 ± 6,0 **	68,7 ± 23,0	50,6 ± 13,7	52,9 ± 15,3	146,9 ± 23,1	64,2 ± 14,2	28,6 ± 3,4
**8 weeks**	25,0 ± 2,6 **	51,2 ± 6,7	64,7 ± 4,4 *	31,3 ± 3,0	204,4 ± 17,7 **	63,2 ± 4,7 **	58,4 ± 5,7
**16 weeks**	23,9 ± 2,3 **	100,3 ± 9,1	110,8 ± 11,0	90,2 ± 12,7	116,5 ± 21,1	80,6 ± 7,7	75,9 ± 7,1
**PLASMA**							
	**4β-OHC**	**5α,6α-epoxyChol**	**5β,6β-epoxyChol**	**7-OHC**	**7-OHCnone**	**24(*S*)-OHC**	**27-OHC**
**1 week**	61,5 ± 4,6 **	88,8 ± 5,8	90,6 ± 8,2	113,0 ± 7,7	116,7 ± 14,5	98,8 ± 7,1	101,4 ± 7,6
**2 weeks**	55,5 ± 1,8 **	83,3 ± 5,3	88,6 ± 5,0	108,6 ± 5,6	192,1 ± 22,4 *	84,5 ± 2,9	90,3 ± 5,0
**4 weeks**	55,5 ± 2,7 **	93,3 ± 12,0	82,3 ± 5,6	103,0 ± 8,7	118,3 ± 21,1	86,4 ± 5,6	107,3 ± 6,7
**6 weeks**	51,5 ± 2,1 **	79,6 ± 5,3	79,5 ± 3,9	78,7 ± 4,9	184,8 ± 13,7 *	77,5 ± 4,3 *	90,3 ± 5,9
**8 weeks**	48,9 ± 2,5 **	84,0 ± 5,8	79,4 ± 5,1	62,7 ± 6,1	147,4 ± 15,0	82,4 ± 1,9 *	111,2 ± 5,5
**16 weeks**	27,4 ± 1,7 **	68,2 ± 4,0 **	62,6 ± 5,1	68,2 ± 10,7	91,0 ± 15,6	54,1 ± 2,1 **	76,5 ± 2,2 **

Table S4. Hepatic oxysterol in genetic models normalized to cholesterol content and relative to respective CTL

Oxysterol levels (pmol/mg of cholesterol) in the liver, adipose tissue and plasma in the genetic models of obesity normalized to the levels found in the respective lean control group. Data are mean ± s.e.m.; student's t-test between ob/ob group vs ob/lean group or db/db group vs db/lean group *P<0,01 and **P<0,001.

ob/ob							
	**4β-OHC**	**5α,6α-epoxyChol**	**5β,6β-epoxyChol**	**7-OHC**	**7-OHCnone**	**25-OHC**	**27-OHC**
**Liver**	13,4 ± 1,6 *	13,3 ± 4,6	11,1 ± 2,9	10,6 ± 3,4	43,4 ± 13,0	25,9 ± 3,0	50,1 ± 7,3
**Adipose tissue**	26,1 ± 1,9	77,7 ± 15,6	68,2 ± 8,3	64,0 ± 5,8 *	49,5 ± 5,8	39,4 ± 3,7	41,9 ± 2,4
**Plasma[1]**	83,4 ± 3,6	91,6 ± 12,4	84,2 ± 12,0	74,6 ± 13,3	105,1 ± 9,2	82,1 ± 3,7	101,0 ± 3,2
**db/db**							
	**4β-OHC**	**5α,6α-epoxyChol**	**5β,6β-epoxyChol**	**7-OHC**	**7-OHCnone**	**25-OHC**	**27-OHC**
**Liver**	63,9 ± 16,2	32,3 ± 7,9 **	45,7 ± 9,7 *	49,5 ± 10,4 *	176,8 ± 81,2	56,5 ± 7,7	318,3 ± 58,8

[1] For plasma 24(*S*)-OHC instead of 25-OHC

Table S5. Composition of the diets used in the diet-induced obesity model

Percentage of energy (kcal) provided by the fat, carbohydrates and proteins and fatty acid composition, of the diets according to the manufacturer.

	CTL (AIN-93M)	HFD (D12492)
**kcal from food**		
Fat	9,4	60
Carbohydrates	75,9	20
Proteins	14,7	20
**Fatty Acids**		
Saturated (%)	14,2	32
Monounsaturated (%)	24,3	35,9
Polyunsaturated (%)	61,4	32
**Acyl chain lenght and insaturation**		
C10	0	0,1
C12	0	0,2
C14	0	2,8
C15	0	0,2
C16	4,16	49,9
C16:1	0	3,4
C17	0	0,9
C18	1,52	26,9
C18:1	9,72	86,6
C18:2	21,4	73,1
C18:3	3,12	5,2
C20	0	0,4
C20:1	0	1,5
C20:2	0	2
C20:3	0	0,3
C20:4	0	0,7
C22:4	0	0
C22:5	0	0,2
C22:6	0	0
C24	0	0
C24:1	0	0
Total	39,92	254,5
